# Integration of job satisfaction into the Personal Wellbeing Index: analysis of psychometric properties and sociodemographic influences among employees of the Croatian Health Insurance Fund

**DOI:** 10.2478/aiht-2025-76-4060

**Published:** 2025-12-30

**Authors:** Jozo Dubravac, Renata Barić

**Affiliations:** University of Zagreb Faculty of Kinesiology, Zagreb, Croatia

**Keywords:** Croatia, occupational health, quality of life, resilience, spillover effect, subjective wellbeing, učinak prelijevanja, Hrvatska, kvaliteta života, otpornost, subjektivna dobrobit, zdravlje na radu

## Abstract

This study examines subjective wellbeing (SWB) among administrative staff of a Croatian universal health insurance coverage provider in a transitional economy, hypothesising a moderate spillover effect from job satisfaction to overall life satisfaction. Our aim was to assess the psychometric properties of a modified Personal Wellbeing Index that incorporates job satisfaction (PWI-J), evaluate sociodemographic influences, and validate the job satisfaction domain inclusion in the index. The hypothesis was tested on a convenience sample of 1,051 Croatian employees of the Croatian Health Insurance Fund who completed an anonymous online survey in 2018. Analyses encompassed descriptive statistics, reliability testing, correlations, ANOVA/MANOVA for sociodemographic effects, and exploratory factor analysis (EFA). The PWI-J exhibited high reliability (Cronbach’s α=0.896), and its two-factor structure (material-practical and socioemotional wellbeing) explained 65 % of the variance. The mean PWI-J score was 65.01 on a 0–100 scale. Standard of living and future security received the lowest score (around 52) and relationships and community the highest (77–81). Spillover was confirmed (ρ=0.633, P<0.001). Compared to the Australian wellbeing triage ranges, participants reported lower economic satisfaction but stronger community ties, with higher education associated with better material outcomes, female gender with enhanced social satisfaction, and younger workers (19–34 years old) with higher socioemotional scores. Our findings underscore economic vulnerabilities balanced by social resilience and validate PWI-J for occupational health assessments. Recommendations include targeted interventions for lower-educated and older workers. Limitations encompass cross-sectional design, self-report bias, and limited generalisability. Future research should pursue longitudinal studies on possible mediators, such as resilience.

## Abstract (hr)

Ovo istraživanje ispituje subjektivnu dobrobit (SWB) među administrativnim djelatnicima obaveznog zdravstvenog osiguranja u tranzicijskoj ekonomiji, postavljajući hipotezu o učincima prelijevanja (engl. *spillover effects*) sa zadovoljstva poslom na zadovoljstvo životom. Ciljevi su bili procijeniti psihometrijska svojstva izmijenjenog Indeksa osobne dobrobiti (PWI-J, koji uključuje domenu zadovoljstva poslom), procijeniti sociodemografske utjecaje i potvrditi opravdanost uključivanja ove domene. Prigodan uzorak od 1.051 djelatnika Hrvatskog zavoda za zdravstveno osiguranje ispunio je anonimnu *online* anketu 2018. godine. Analize su obuhvatile deskriptivnu statistiku, testiranje pouzdanosti, korelacije, ANOVA/MANOVA za sociodemografske učinke te eksploratornu faktorsku analizu (EFA). PWI-J pokazao je visoku pouzdanost (Cronbachov α=0,896) i dvofaktorsku strukturu (materijalno-praktična i socioemocionalna dobrobit, koje objašnjavaju 65 % ukupne varijance). Prosječni rezultat Indeksa osobne dobrobiti bio je 65,01 na ljestvici od 0 do 100, s najnižim zadovoljstvom u standardu života i sigurnosti u budućnosti (oko 52), a najvišim u odnosima i zajednici (77 – 81). Učinak prelijevanja je potvrđen (ρ=0,633, P<0,001). U usporedbi s australskim normama, ispitanici su pokazali niže ekonomsko zadovoljstvo, ali jače veze sa zajednicom, pri čemu je viša razina obrazovanja povezana s boljim materijalnim ishodima, žene su zadovoljnije s društvenim povezanostima, a mlađi radnici s višim socioemocionalnim rezultatima. Nalazi naglašavaju ekonomske ranjivosti uravnotežene društvenom otpornošću, potvrđujući valjanost PWI-J upitnika za procjenu kvalitete života na poslu. Preporuke uključuju ciljane intervencije za slabije obrazovane i starije radnike. Ograničenja obuhvaćaju poprečni dizajn, pristranost samoprocjene i ograničenu generalizaciju rezultata. U budućim istraživanjima trebale bi se provesti longitudinalne studije na medijatorima poput otpornosti na stres.

Unlike quality of life (QoL) assessments which include objective indicators, such as income or physical health, subjective quality of life (sQoL) assessments focus on personal perceptions shaped by cultural and social contexts (1). One such subjective cognitive and emotional assessment that includes life satisfaction, happiness, and a sense of purpose is the so called subjective wellbeing (SWB) (2). However, for the sake of conceptual disambiguation it is important to distinguish SWB from sQoL, even though these terms are often used interchangeably in the literature. SWB is primarily concerned with emotional and cognitive evaluation of one’s life, whereas sQoL encompasses a broader range of life domains, including subjective perception of objective factors. It is usually measured with the Personal Wellbeing Index for adults (PWI-A), which focuses on satisfaction across life domains, without including affective components of SWB, using tools such as the PhenX Toolkit (3). According to the Personal Wellbeing Index manual, “Subjective Life Quality can only be validly, empirically measured, by asking for an individual’s opinion about their feelings” (4).

The PWI-A has been translated into over 30 languages and validated in Croatia (5). With its Cronbach’s α typically ranging from 0.70 to 0.85 (6, p. 760), the PWI demonstrates high reliability and cross-cultural comparability across different countries (6, pp. 769–770). However, it lacks a specific job satisfaction domain, despite work’s central role in adult life.

Job satisfaction is a key determinant of SWB, as work experiences spill over to other life domains, such as personal relationships and financial stability, and vice versa (7, 8). For example, Unanue et al. (9) have demonstrated that fulfilling psychological needs at work – autonomy, competence, and relatedness – enhances both job satisfaction and overall life satisfaction. Studies confirm that job satisfaction mediates SWB, particularly in high-stress professions, where burnout risks are elevated (10, 11). The guidelines issued by the Organisation for Economic Co-operation and Development (OECD) emphasise job satisfaction as a core component of SWB, supporting its inclusion in wellbeing assessments (12). We therefore believe that integrating job satisfaction into the PWI-A could enhance its applicability to working populations, as it addresses a critical gap in existing measures. Its inclusion into wellbeing assessments is further justified by the use of other tools like the Quality of Life Inventory (13) and by established research which identifies work as a key life domain and a fundamental determinant of overall quality of life (14).

For administrative workers in the public health insurance sector, who face emotional demands and ergonomic challenges, a tool like the PWI-A with the integrated job satisfaction domain (PWI-J, with “J” standing for “job”) could standardise SWB in a working population. In Croatia, where economic challenges contrast strong social resilience, such tools support the development of integrated occupational health strategies (15). The PWI-J can guide occupational health professionals in designing stress management programmes, improving work-life balance, and shaping policies to reduce employee turnover, thereby contributing to more productive and resilient work environments.

## Study objectives

The aims of this study were to address the current gap in SWB measurements and to test the validity of the newly developed PWI-J questionnaire among administrative workers in the national public health insurance service. The specific objectives were: a) to verify the psychometric properties (internal consistency, factor structure) of the PWI-J using exploratory factor analysis to investigate its underlying structure in this novel context; b) to examine sociodemographic influences (age, gender, education) on the relationship between job satisfaction and SWB; and c) to validate the inclusion of job satisfaction through convergent validity and reliability analyses.

Our hypotheses were as follows:

H1: The PWI-J will demonstrate strong psychometric properties, including high internal consistency and a clear factor structure where job satisfaction loads significantly on a relevant factor.
H2: Job satisfaction will show a moderate to strong positive correlation with overall PWI-A scores, supporting the spillover effect.
H3: Sociodemographic factors, namely higher education, female gender, and younger age, will be associated with higher levels of wellbeing in their respective domains.

## PARTICIPANTS AND METHODS

### Participants

Between October and November 2018, we recruited via email a convenience sample of 1,067 workers of the Croatian Health Insurance Fund – the central national insurance organisation overseeing the implementation of universal healthcare coverage – who were based in the nation’s capital Zagreb. Following the exclusion of 16 respondents who provided consistently extreme scores (0 or 10) across all domains – a step taken to ensure response validity as recommended in PWI validation studies (16) – the final sample included 1,051 participants. These individuals held a variety of professional roles within the health insurance system, including medical and legal professionals, administration and finances. We believe that data collected in 2018 provide a pre-COVID baseline for understanding wellbeing trends, which are particularly relevant given the subsequent pandemic’s impact on healthcare-related administrative workers. Such a baseline is essential for a comparative longitudinal research, allowing future studies to track changes in subjective wellbeing over time and to assess the long-term effects of the pandemic on healthcare-related administrative workers in transitional economies like Croatia.

The sample comprised 853 women (81.2 %) and 198 men (18.8 %), reflecting the healthcare sector’s gender distribution, though this imbalance may limit the generalisability of gender-based analyses. In terms of education 532 (50.6 %) had a secondary level, 129 (12.3 %) a bachelor’s or higher vocational degree, and 390 (37.1 %) held a master’s degree.

The sample size was sufficient to detect medium effect sizes (η^2^=0.06) with 80 % power at α=0.05 for the analysis of variance (ANOVA), multivariate analysis of variance (MANOVA), and factor analyses, as reported by earlier PWI-A studies (17, 18).

### Instrument

Subjective wellbeing (SWB) was assessed with the newly developed PWI-J instrument combining the Croatian version of the PWI-A, standardised by Kaliterna Lipovčan and Prizmić-Larsen (18) and validated by Kaliterna Lipovčan et al. (5), and the newly added job satisfaction domain to the original seven domains: standard of living, health, achievements, relationships, safety, community connectedness, and future security. The eighth domain, job satisfaction, was tailored to assess psychosocial factors relevant to the quality of life of public sector workers. Each domain was rated on an 11-point Likert scale (0 – completely dissatisfied to 10 – completely satisfied) converted to a 0–100 scale.

The survey was completely anonymous and confidential and was approved by their employer, the Croatian Health Insurance Fund in accordance with Croatian regulations governing research in public-sector organisations that lack a dedicated ethics committee. All procedures were performed in full compliance with the Declaration of Helsinki.

Participants were informed that there were no correct or incorrect answers, and data were analysed at an aggregate level, preventing individual identification.

### Statistical analysis

Normality of distribution was assessed as the first step in evaluating assumptions for parametric testing. Given the large sample size (N=1,051), Kolmogorov-Smirnov tests were significant for all items (P<0.001), which was attributed to high statistical power rather than meaningful deviations from normality (19). Normality was therefore judged primarily using skewness and kurtosis statistics. All values (|skewness|≤1.33; |kurtosis|≤1.64) fell well within the recommended thresholds (<2 and <7, respectively) for robust parametric analyses in large samples (20). Based on this evidence, we used one-way ANOVA (with education level, gender, or age group as the independent variable, depending on the hypothesis tested) for between-group comparisons of PWI-J domain and composite scores. Post-hoc comparisons were done using the Tukey’s honestly significant difference (HSD) test to control for the family-wise error rate.

## RESULTS

Table 1 shows the distribution of study participants by sociodemographic characteristics, and Table 2 their scores for the eight PWI-J domains. Overall, the relationships and community domains received the highest, while standard of living and future security the lowest scores.

**Table 1:** Distribution of study participants by sociodemographic characteristics (N=1,051)*

**Characteristic**	**Category**	**Frequency (N)**	**Percentage (%)**
Gender	Female	853	81.20
	Male	198	18.80
Age group	19–34	196	18.60
	35–44	367	34.90
	45–54	297	28.30
	55 and over	191	18.20
Education level	Secondary education	532	50.60
	Bachelor’s degree	129	12.30
	Master’s degree	390	37.10

* after exclusion of 16 participants over nullified questionnaires

**Table 2:** PWI-J scores and distribution by domains (N=1,051)

**PWI domain**	**Min**	**Max**	**Mean**	**SD**	**Skewness**	**Skewness SE**	**Kurtosis**	**Kurtosis SE**
Standard of living	0	100	52.22	25.10	−0.209	0.075	−0.559	0.151
Health	0	100	67.32	23.63	−0.665	0.075	−0.079	0.151
Achievements	0	100	65.32	21.99	−0.552	0.075	−0.110	0.151
Relationships	0	100	81.33	19.23	−1.325	0.075	1.636	0.151
Safety	0	100	63.66	25.96	−0.636	0.075	−0.389	0.151
Community	0	100	77.35	20.00	−1.141	0.075	1.120	0.151
Future security	0	100	52.35	28.01	−0.237	0.075	−0.924	0.151
Job satisfaction	0	100	60.49	26.17	−0,491	0.075	−0.541	0.151
PWI-A (without job satisfaction)	4	100	65.65	17.08	−0.593	0.075	0.199	0.151
PWI-J (job satisfaction included)	5	98.75	65.01	17.20	−0.555	0.075	0.099	0.151

Scale 0–100; SD – standard deviation; SE – standard error

Our findings confirm all three hypotheses, as follows.

### Internal consistency and domain correlations

The internal consistency of the PWI-J was high, with Cronbach’s α=0.896 indicating high reliability. Corrected single domain to total correlations were satisfactory, ranging from 0.492 (relationships) to 0.729 (safety). Although the correlation for the relationships domain was relatively low, it was above the conventional threshold of >0.40. The reliability analysis further supported the inclusion of the job satisfaction domain, as its deletion would lower the scale’s α to 0.848 (Table 3).

**Table 3:** Domain-to-total-score correlations for PWI-J

**PWI domain**	Mean* if domain deleted	**Variance if domain deleted**	**Corrected domain to total score correlation**	Squared multiple correlation**	**Cronbach’s alpha if domain deleted**
Standard of living	66.83	14,609	0.610	0.480	0.851
Health	64.68	15,342	0.519	0.295	0.861
Achievements	64.96	14,717	0.701	0.527	0.842
Relationships	62.67	16,164	0.492	0.402	0.862
Safety	65.20	13,817	0.729	0.600	0.836
Community	63.24	15,515	0.607	0.476	0.852
Future security	66.81	13,754	0.670	0.547	0.844
Job satisfaction	65.65	14,300	0.632	0.436	0.848

* Mean – average score of the 0–100 scale excluding the item.

** Squared multiple correlations – the proportion of item variance explained by other items

Pearson correlation analysis shows that all inter-domain correlations, as well as the correlations of each domain with the total PWI-J score are positive and statistically significant (P<0.001) (Table 4). The strongest correlations with the PWI-J total score are those for safety (r=0.811), achievements (r=0.777), and future security (r=0.774). The highest inter-domain correlations are those between safety and future security (r=0.685) and between community and relationships (r=0.578), whereas the lowest are between relationships and standard of living (r=0.217).

**Table 4:** Pearson’s correlation coefficients between the PWI-J domains

**PWI domain**	**Standard of living**	**Health**	**Achievements**	**Relationships**	**Safety**	**Community**	**Future security**	**Job satisfaction**
Standard of living	1							
Health	0.365^**^	1						
Achievements	0.594^**^	0.459^**^	1					
Relationships	0.217^**^	0.378^**^	0.458^**^	1				
Safety	0.508^**^	0.398^**^	0.522^**^	0.357^**^	1			
Community	0.300^**^	0.420^**^	0.439^**^	0.578^**^	0.525^**^	1		
Future security	0.566^**^	0.348^**^	0.531^**^	0.304^**^	0.685^**^	0.431^**^	1	
Job satisfaction	0.489^**^	0.355^**^	0.506^**^	0.335^**^	0.593^**^	0.446^**^	0.465^**^	1
PWI-J	0.718^**^	0.639^**^	0.777^**^	0.594^**^	0.811^**^	0.695^**^	0.774^**^	0.739^**^

** P<0.001 (2-tailed)

### Spillover effect

Job satisfaction and the overall PWI-J score show a strong Spearman’s correlation (ρ=0.633, P<0.001; applied due to non-normal data distribution). Given the cross-sectional design, causal direction cannot be inferred. However, this association is consistent with the spillover hypothesis, which suggests that experiences in one central life domain, such as work, can influence perceptions of overall life satisfaction (7, 8, 21).

### Sociodemographic differences in PWI-J domains

We tested our third hypothesis of the association between sociodemographic factors and PWI-J domains with a series of ANOVAs (Table 5). Education level shows significant effects on the standard of living, health, achievements, and future security, with the post-hoc Tukey’s tests indicating higher scores among higher-educated participants. Gender showed significant effects on achievements, relationships, safety, community, and job satisfaction, with women reporting higher satisfaction. Age showed significant effects on health, relationships, safety, community, and job satisfaction, with younger participants (19–34-year-olds) generally reporting higher satisfaction than older groups.

**Table 5:** ANOVA F ratios and significance for PWI-J domains by sociodemographic factors

**PWI domain**	**Gender F (1,1027)**	**Education F (2,1027)**	**Age F (3,1027)**	**Gender × Education F (2,1027)**	**Gender × Age F (3,1027)**	**Education × Age F (6,1027)**	**Gender × Education × Age F (6,1027)**
Standard of living	1.82	16.62^***^	2.20	0.92	1.03	2.67	2.12
Health	2.85	6.30^**^	8.05^***^	1.53	0.69	1.05	0.53
Achievements	7.57^*^	13.65^***^	0.34	2.03	1.17	1.52	1.22
Relationships	6.77^*^	0.73	3.00^*^	2.99	2.47	0.62	0.67
Safety	4.41^*^	2.68	3.30^*^	1.12	1.66	2.27^*^	1.89
Community	4.34^*^	0.51	3.41^*^	0.23	1.65	1.85	0.86
Future security	2.67	11.96^***^	0.33	1.67	0.72	1.44	0.90
Job satisfaction	8.10^***^	2.30	2.72^*^	2.37	0.76	2.60	1.22

Note:

* P<0.05,

** P<0.01,

*** P<0.001.

F – ratio from two-way and three-way ANOVAs

### Factor structure of the PWI-J

The data proved suitable for the exploratory factor analysis (EFA), as indicated by the Kaiser-Meyer-Olkin measure of sampling adequacy of 0.861 and the significant Bartlett’s test of sphericity [χ^2^(28)=3593.39, P<0.001]. We applied principal component analysis with Promax rotation, given the expected correlation of factors, which yielded two components with eigenvalues exceeding 1. These account for 65 % of the total variance (Table 6).

**Table 6:** Major factors (dimensions) identified with principal component analysis

**Component**	**Initial eigenvalues**			**Extraction sums of squared loadings**			Rotation sums of squared loadings^*^
	**Total**	**% of variance**	**Cumulative %**	**Total**	**% of variance**	**Cumulative %**	**Total**
**Factor 1** Material-practical wellbeing	4.173	52.158	52.158	4.173	52.158	52.158	3.806
**Factor 2** Socioemotional wellbeing	1.027	12.841	64.999	1.027	12.841	64.999	3.046

* When components are correlated, sums of squared loadings cannot be added to obtain a total variance

The pattern matrix (Table 7) highlights a clear two-factor structure: Factor 1, explaining 52.2 % of the variance, shows strong loadings from the standard of living (0.945), achievements (0.625), safety (0.747), future security (0.844), and job satisfaction (0.664) domains. These domains capture material and practical wellbeing, reflecting the aspects tied to economic stability and security.

**Table 7:** Rotated component matrix obtained with principal component analysis

**Domain**	**Factor 1**	**Factor 2**
Standard of living	0.945	-
Health	-	0.523
Achievements	0.625	-
Relationships	-	0.965
Safety	0.747	-
Community	-	0.799
Future security	0.844	-
Job satisfaction	0.664	-

Rotation method: Promax with Kaiser normalisation (with suppressed loadings of <0.30)

Factor 2, accounting for 12.8 % of the variance, shows high loadings from the health (0.523), relationships (0.965), and community (0.799) domains and, being focused on interpersonal and health-related dimensions, represents socioemotional wellbeing.

### Sociodemographic effects on material security and socioemotional wellbeing

In line with the EFA, the PWI-J domains were grouped into two composite scores: material security (averaging standard of living, achievements, safety, future security, and job satisfaction) and socioemotional wellbeing (averaging health, relationships, and community) to establish overall wellbeing patterns. The effects of gender, education level, and age on these two dimensions were tested with the MANOVA.

Box’s M test indicated a violation of the homogeneity of covariance assumption (M=126.34, P<0.001), but given the large sample size (N=1,051), the results are considered robust (22). Levene’s test indicated heterogeneity of variance for material security (P=0.020) but not for socioemotional wellbeing (P=0.058).

The effects of all three sociodemographic factors were significant, as follows: gender [Wilks’ Λ=0.991, F(2, 1026)=4.42, P=0.012, partial η^2^=0.009], education level [Wilks’ Λ=0.975, F(4, 2052)=6.40, P<0.001, partial η^2^=0.012], and age [Wilks’ Λ=0.969, F(6, 2052)=5.40, P<0.001, partial η^2^=0.015]. All effect sizes were small, and we observed no interaction effects (all P>0.05).

Follow-up univariate analyses of variance indicated that gender had an effect on both material security [F(1, 1027)=7.14, P=0.008, partial η^2^=0.007] and socioemotional wellbeing [F(1, 1027)=6.96, P=0.008, partial η^2^=0.007], as women reported higher scores on both: (59.25 vs 56.89 for material security and 75.63 vs 74.06 for socioemotional wellbeing).

Education level affected material security [F(2, 1027)=12.40, P<0.001, partial η^2^=0.024] and participants with graduate or higher education scored higher than those with secondary education (63.95 vs 55.42, P<0.001) or undergraduate education (57.26, P=0.003).

Age had an effect on socioemotional wellbeing [F(3, 1027)=6.13, P<0.001, partial η^2^=0.018], and the youngest age group of 19–34 years reported higher scores than the 45–54 group (80.14 vs 73.39, P<0.001) or 55 and over (72.72, P<0.001).

### Comparison of PWI-J score distribution with the Australian wellbeing triage ranges

Compared with the Australian wellbeing triage ranges (4), the Croatian sample showed a markedly smaller proportion of participants in the “Well” category (70–100 points: normal homeostatic control) and a substantially higher proportion in the “UnderWell” (50–69 points: challenged homeostatic control) and “NoWell” (0–49 points: defeated homeostatic control) categories (Table 8). The higher prevalence of “UnderWell” and especially of “NoWell” scores indicates significant psychosocial challenges and homeostatic defeat in this population.

**Table 8:** Distribution of the PWI-A scores in the 2018 Croatian sample vs. Australian norms

**Range**	**Description**	**Croatian sample % (N=1,051)**	**Australian norms % (N=73,560)**
Well (normal homeostatic control) (≥70 points)	Person can maintain subjective well-being	45.6 % (479)	81.37 % (59,850)
UnderWell (challenged homeostatic control) (50–69 points)	Person may be compromised in maintaining subjective well-being	38.1 % (400)	16.89 % (12,430)
NoWell (defeated homeostatic control) (0–49 points)	Person unable to maintain subjective well-being; intervention may help	16.4 % (172)	1.74 % (1,280)

Percentages refer to the rounded sample (N=1,050). Australian data were taken from (4)

## DISCUSSION

Our study provides valuable insights into subjective wellbeing of public health insurance workers in Croatia, and the adapted PWI-J version, which includes job satisfaction, better captures the occupational context thanks to a robust instrument structure. Two distinct dimensions – material-practical wellbeing and socioemotional wellbeing – stand out as its relative strength, accounting for 65 % of the total variance. This pattern is consistent with the homeostatic theory, which posits that SWB is actively maintained within a set range for most individuals, even in the face of external pressures. The following subsections interpret these and other key results in light of the existing literature.

### Psychometric properties and factor structure

Our PWI-J shows excellent internal consistency (α=0.896), well above the conventional threshold, and falls in line with reliability estimates reported internationally (4, 16, 23, 24). All domain-to-total-score correlations are satisfactory, supporting the inclusion of job satisfaction, particularly in occupational contexts where employment permeates multiple life domains (25).

The two dimensions accounting for 65 % of the variance, namely, material-practical and socioemotional wellbeing, extend the theoretical unidimensional model of the PWI-A often supported in homogeneous Western samples like Australia (4, 26). Cross-cultural studies, however, frequently report more complex structures, particularly in transitional contexts, where economic and security concerns may form a distinct dimension (23, 27). The moderate correlation between the two factors supports their interpretation as related yet distinct components of a higher-order SWB construct.

### Descriptive profile and homeostatic interpretation

The overall mean score for the first seven domains (job satisfaction excluded) of ~65 places our sample in the “UnderWell” group (50–69 points), indicating a state of homeostatic challenge where external pressures have lowered SWB below its normal set-point (70–100 points) but has not resulted in full homeostatic defeat (“NoWell”: 0–49 points) (4). The notably low satisfaction scores with the standard of living and future security likely act as primary “homeostatic attackers”, which is consistent with the reports on populations experiencing economic strain or transitional stressors (60–70 points) (18). In contrast, the relatively high scores in the relationships and community domains may function as key internal and external buffers, helping to mitigate these negative pressures and maintain a degree of stability (29, 30). Furthermore, as stated in the PWI-A manual, providing domain-relevant services to individuals in the “UnderWell” ranges – especially to those scoring below 50 points – has a high probability of raising SWB (4, p. 3–9).

### Domain interrelations and spillover effects

Inter-domain correlations were all positive and significant, with the strongest associations between safety and future security and between community and relationships, whereas the weakest correlation was between relationships and the standard of living, suggesting that social and economic domains operate somewhat independently in our sample.

The spillover effect between general wellbeing (PWI-A) and job satisfaction was also confirmed, supporting the notion that work experiences significantly influence but do not fully determine overall personal wellbeing (7, 9, 21). According to Diener et al. (1), SWB encompasses satisfaction with key domains such as work, which has been confirmed by a meta-analysis on the bidirectional relationship between job satisfaction and SWB (8). Iqbal et al. (31) emphasise that work-life balance acts as a mediator, with intrinsic motivation as a moderator. In the context of contemporary challenges such as hybrid work and automation (32), incorporating job satisfaction into the PWI-A enhances its applicability to the working population, as evidenced by our results and supported by Kuykendall and Tay (33).

### Cross-cultural comparison

Compared to Australian triage ranges (4), the Croatian sample shows a substantially smaller proportion of participants in the “Well” range. This divergence underscores the influence of macroeconomic and socio-political context on population wellbeing and cautions against an uncritical application of norms derived from stable, high-income countries (24).

### Sociodemographic variations

Sociodemographic factors exerted statistically significant yet modest effects, suggesting that they can explain a limited portion of variance in SWB compared to other contextual factors.

Higher education was associated with greater satisfaction in material and security-related domains, reinforcing its role as a resource buffer (34).

Women reported higher satisfaction than men across several domains, particularly those related to social connectedness and safety. Although this gender pattern is frequently observed in Western European samples (35), studies in Croatia and other post-Yugoslav countries have yielded mixed or context-specific findings. Our finding of higher satisfaction of women in social domains may reflect the enduring influence of collectivist cultural norms and stronger family/community ties in transitional societies recovering from the conflicts in the 1990s. These are the factors that appear to buffer women more effectively against economic stressors than men (6, 23, 36).

Younger participants generally reported higher socioemotional wellbeing, possibly reflecting cohort effects or life-stage advantages in social engagement and health (37, 38).

### Study limitations

The cross-sectional design precludes causal inferences, as it captures wellbeing at a single point in time without tracking changes over the years. This is particularly relevant, given that the data were collected in 2018, prior to the COVID-19 pandemic, which has since profoundly impacted workers’ stress levels and subjective wellbeing globally. Longitudinal studies would therefore be essential to examine how these patterns evolve.

Another design limitation is that the measures are self-reported and may introduce biases, such as social desirability or recall inaccuracies, which could inflate correlations between domains like job satisfaction and overall SWB.

The third is convenience sampling, as it may have led to selection bias. This resulted in a sample skewed toward women (81.2 %), residents of the Croatian capital, and public sector, limiting generalisability to other regions, private sector workers, or more diverse demographic groups in Croatia.

Finally, although sociodemographic factors were analysed, we did not control for additional confounders like work tenure or shift patterns, which may have influenced the observed effects.

Despite these limitations, the study’s strengths in psychometric validation and contextual relevance provide a solid foundation for future research in transitional economies.

## CONCLUSIONS

In summary, this study supports the validity and reliability of the PWI-J in Croatian occupational context. The results are consistent with the homeostatic theory, depicting a population under significant economic pressure, partly buffered by strong relational and community resources. The observed two-factor structure suggests that wellbeing in this context is cognitively organised along material-practical and socioemotional lines, a nuance that may inform culturally sensitive intervention strategies. For instance, workplace interventions should target both material security (e.g., fair compensation) and socioemotional support (e.g., team-building) to effectively enhance overall wellbeing. Future research should explore the longitudinal stability of these factors and their interaction with specific workplace conditions and national economic trends.

Ultimately, this work underscores that wellbeing is not a one-size-fits-all concept but is deeply woven into cultural and economic contexts. Croatia’s combination of resilient social bonds and economic challenges offers a valuable perspective for refining subjective wellbeing theories in contemporary society.
